# Using the theory of positive disintegration’s ‘dynamisms’ to gauge retrospective suicide lived experience

**DOI:** 10.3389/fpsyg.2026.1810469

**Published:** 2026-06-17

**Authors:** Marie-Lise Schläppy, Michael J. Kyron, Susanne H. Stanley, Dawn S. Freshwater, Sean D. Hood

**Affiliations:** 1 UWA Medical School, Psychiatry, The University of Western Australia, Perth, WA, Australia; 2 School of Psychological Science, University of Western Australia, Perth, WA, Australia

**Keywords:** guilt, level III, dynamisms, shame, suicide, suicide prevention, theory of positive disintegration

## Abstract

In this study, the concept of ‘disintegration’, as explained in the Theory of Positive Disintegration (TPD) was tested to find out whether it could be used as an indication of retrospective suicide lived experience. This study constitutes one of the first baseline of TPD level III ‘dynamisms’ in a sample of participant with(out) suicide lived experience. The aims of this study were to test, via an online survey, whether TPD level III disintegrative ‘dynamisms’ were associated with the retrospective behaviours, emotions, or thoughts of study participants with(out) suicide lived experience. Two Kruskal-Wallis median tests showed that disintegrative ‘dynamisms’ were (a) significantly higher in participants with suicide lived experience and (b) were higher in persons supported pharmacologically for their mental health. To investigate (a) the ‘dynamisms’ themselves, (b) their location (i.e., behaviour, thoughts or emotions) and how far in the past they occurred (months or years), a PERMANOVAs and non-metric multi-dimensional scaling (nMDS) based on Euclidean distances were carried out. Those multivariate analyses showed that the different level III ‘dynamisms’ were statistically different to each other and therefore not interchangeable. The location of ‘dynamisms’ mattered, and participants scored them differently for behaviour, thoughts and emotions. Unlike other ‘dynamisms’, ‘shame’ and ‘guilt’ elicited the same responses from participants whether the ‘dynamisms’ were months or years in the past, suggesting that those two ‘dynamisms’ may be the best candidates for counsellors to use to address disintegration and suicide prevention with their patients. Overall, the study results suggests that there is merit in using the vocabulary of TPD ‘dynamisms’ in suicide prevention.

## Introduction

Suicide remains a significant global health issue and a leading cause of death amongst individuals aged 15–29 years, with 720,000 people of all ages dying each year (WHO, 2025). The lived experience of suicidal distress is complex and multi-dimensional. Our ability to recognise how people express internal distress remains limited, and reliable early-detection methods are scarce; even trained health professionals struggle to predict it consistently (Bryan and Rudd, 2006). The continued occurrence of deaths by suicide underscores the urgent need to continue refining our understanding of the internal psychological forces at play.

Using existing frameworks offer the possibility of characterising distress experienced with suicide lived experience. For example, the Theory of Positive Disintegration (TPD) conceptualises disintegration as a state of internal psychological turmoil which, if unresolved, may increase the risk of suicide (Dąbrowski, 1964). Central to TPD are ‘dynamisms’, which are specific emotional and cognitive experiences that signal disintegration, including ‘shame’, ‘guilt’, ‘self-disappointment’, ‘astonishment’ or ‘surprise with oneself’, ‘worry about the self’, and feelings of ‘inferiority’ (Dąbrowski, 1964). While some of these experiences, such as ‘guilt’ and ‘self-disappointment’, are integrated into many standardised tools, others, such as ‘astonishment’ or ‘surprise with self’, remain largely unexamined in the context of mapping internal states which may represent important, early indicators of internal distress. As such, TPD’s vocabulary of ‘dynamisms’ could offer a more nuanced way to engage individuals in conversations about their psychological state, potentially serving as a gentle entry point for assessing distress before asking direct questions about suicidal thoughts or behaviours and before offering self-report scales.

### Theory of Positive Disintegration (TPD)

The Theory of Positive Disintegration (TPD) was elaborated by K. Dąbrowski (1902–1980) (Dąbrowski, 1964; Dąbrowski et al., 1970) and posits that there are five levels of personality development (Dąbrowski, 1964; Schläppy, 2019). Unlike conventional wisdom, TPD proposes that symptoms such as depression and anxiety present at certain levels, could be positive in certain circumstances, and could be a way to attain a higher level of personality development, not merely pathology. According to TPD, only when the disintegrative state pertains overtime, does it become negative disintegration (Dąbrowski, 1964).

At the heart of TPD are ‘dynamisms’ which are forces that create tension between an individual’s current inner state and their values. Those ‘dynamisms’ are thought to be the forces that propels an individual from one level to another (Dąbrowski, 1964; Dąbrowski et al., 1970). They have been described as “instincts, drives, and intellectual processes combined with emotions” (Dąbrowski, 1972a, p. 294) and they relate to the transition to another level.

This study is, to our knowledge, one of the first attempts at empirically mapping a person’s inner retrospective inner state using TPD ‘dynamisms’ and at empirically describing how these ‘dynamisms’ occur in the general population.

### Study aims

This study assesses whether participants with suicide lived experience (defined here as: suicidal ideation, making plans, or acting on those plans) are more likely to have experienced high degrees of level III ‘dynamisms’. The aims in this study are to carry out an innovative exploratory study of retrospective inner states relating to suicide to empirically (in)validate whether the wording of TPD’s ‘dynamisms’ could be used to characterise the distressed states linked to suicide lived experience.

Therefore, the following hypotheses were tested:

1. Survey participants with suicide lived experience self-report higher retrospective degrees of disintegration compared to a control group without suicide lived experience.
2. The retrospective effect of a ‘dynamism’ is not equal across all ‘dynamisms’ types.
3. Medication for mental health lowers retrospective disintegration scores measured through ‘dynamisms’.
4. ‘Dynamisms’ have a specific ‘location’ (i.e., a person’s behaviour, thoughts, or emotions).
5. Disintegrative ‘dynamisms’ are experienced over months or years.

## Methods

Study participants were recruited through a paid survey service and, if they were >18 years old, they were offered the opportunity to fill out an online survey. The service ensured that at least 300 participants completed the questionnaire, and that no same person filled the questionnaire multiple times (Supplementary file S2).

The survey questionnaire (Supplementary file S2) was designed to take an average of 20 min to complete. Data from participants were excluded from analysis if (1) the participant took less than seven minutes to complete the survey and if (2) they wrote nonsensical answers in a compulsory field. This left 262 participants with valid responses.

The questionnaire consisted of 36 questions on a 7-point Likert scale (S1). The questionnaire was split into sections and separated by two ‘well-being questions’ to check if the participants were well enough to continue taking the survey.

Participants were asked whether they had had suicide lived experience. If they did, they could describe their lived experience in an open field (not compulsory). For those who did describe their suicide lived experience, their answer was later categorised as one of four categories: ‘ideation’, ‘making plans’, ‘acting on plans’, and ‘unspecified’ (Table 1). Participants with suicide lived experience (i.e., treatment group) were instructed to base their answers on that experience. The participants without suicide lived experience (i.e., control group) were asked to recall a difficult life event instead, to anchor their responses (e.g., loss of a loved one/ serious medical diagnosis). Since it was not possible to verify if individuals in the control group had unreported histories of suicidal thoughts or behaviours, their responses were accepted at face-value.

**Table 1:** Participants were assigned to different groups depending on whether they had suicide lived experience or not.

Event type	Lived experience type	Group name
Suicide lived experience	Ideation	Ideated
	Making plans	Planned
	Acted on the plans	Acted
	Unspecified	Unspecified
Other than lived experience	x	Control

If they had chosen to describe their lived experience in an open field, the information became codified into the ‘ideation’, ‘making plans’ or ‘acted on the plans’ lived experience types. The answers of participants who declined to answer the open field were placed into the ‘unspecified’ category.

Questionnaire items were constructed using the same ‘dynamisms’ vocabulary found in TPD (Dąbrowski, 1972a, 1972b) (Supplementary file S2, P. 253) ‘out of proportion’, ‘guilt’, ‘shame’, ‘surprised’ (with self), ‘worried’, ‘inferior’, ‘dissatisfied (with self) and ‘dissatisfied (as I can) do better’ (S1, Table 2). In addition, the 7-point Likert scale questionnaire included questions about at what ‘Location’ (‘Behaviour’ ‘Thoughts’ or ‘Emotions’ ‘Not applicable (N/A)’) and at what ‘time’ (‘Years’ or ‘Months’) the ‘Dynamism’ was experienced (Table 2). The questions were intended to be exploratory, not psychometric. After checking for Cronbach Alpha and after an exploratory factor analysis to ensure the uni-dimensionality of the items (see statistics below) an overall ‘dynamism’ score was calculated by taking the median across all ‘dynamisms’ categories to obtain an overall measure of disintegration. Participants were also requested to indicate whether they were taking medication to support their mental health: ‘Never’, ‘Now’, ‘In the past’, ‘Now and in the past’.

**Table 2:** Variables used in this study.

Variable type	Variable name	Levels
Dependent	Out of proportion (acting…)	x
	Guilt	x
	Shame	x
	Surprised (with self)	x
	Worried (about self)	x
	Inferior (to others)	x
	Dissatisfied (with self)	x
	Dissatisfied (with self, I know I) can do better	x
Independent	Gender	Female, Male
	Location (of dynamism)	Behaviour, thoughts, emotions, not applicable (N/A)
	Time (of dynamism)	Month, year
	Medicine for mental health	None, ‘Yes, now’, ‘Yes, in the past’, ‘Yes, now and in the past’

Dependent variables were the dynamisms Likert scores (Dynamisms expressed as per the Theory of Positive Disintegration).

### Statistical analyses

A two-pronged statistical approach was used to analyse the data. First, the level of disintegration as given by an overall ‘dynamism’ score was compared across different participants’ characteristics (Table 2, independent variables).

To ascertain whether the overall median ‘dynamism’ score could legitimately be used, and to assess the internal consistency of the 7-point Likert scale 36 survey items, Cronbach’s alpha coefficients were calculated. The 36 items (Supplementary file S2) demonstrated excellent reliability (*α* = 0.984) (Supplementary Table S1). No items were removed as the analysis ‘alpha if item deleted’ showed that values were either *α* = 0.983 or *α* = 0.984 indicating that removing any single item would not have improved the overall consistency of the instrument.

First, an exploratory factor analysis, which was conducted to examine the underlying structure of the items and test the one-dimensionality of the 36 items, yielded a strong unidimensional structure (Supplementary Table S2). The first factor accounted for 63.78% of the total variance, whereas all subsequent factors contributing substantially less (Supplementary Table S2). Inspection of the factor loadings showed that all items loaded strongly on the first factor, with loadings ranging from 0.618 to 0.871 and 22 of them exceeded 0.80 (Supplementary Table S2). These findings suggested that the items primarily reflect a single underlying construct and that it was legitimate to compute a total ‘dynamism’ score for further analyses. No rotation was applied because only one factor was retained. The Kaiser-Meyer-Olkin measure verified sampling adequacy (KMO = 0.939), and Bartlett’s test of sphericity was significant [χ^2^ (630) = 13925.08, *p* < 0.001].

Second, non-parametric analyses were carried out with the statistical software SPSS. To ascertain whether gender needed to be considered in subsequent analyses, an independent-samples Mann–Whitney U test was conducted to determine if there were differences in the overall median ‘dynamism’ scores between genders. This non-parametric approach was selected because the data were Likert scores ranks and the assumptions of normality and homoscedasticity required for parametric testing could not be presumed. This test was chosen over the chi-square test as it provided greater statistical power by using the full rank order of the data to compare the distributions between groups (Agresti, 2010, Siegel and Castellan (1988)).

In addition, a non-parametric Kruskal-Wallis median test was carried out on test scores of participants (with-)out lived experience (Table 1). Pairwise comparison using the Dunn test and the Bonferroni correction and the *α* = 0.05 significance level were carried out between pairs of event types (Table 1). To test where (e.g., ‘Behaviour’, ‘Emotions’, or ‘Thoughts’) and when (‘Months’, ‘Years’) the ‘dynamisms’ were felt, the questions included all those possibilities for each ‘dynamism’, except ‘Shame’ and ‘Guilt’ which lacked a time stamp (S1, Table 2).

A non-parametric Kruskal-Wallis median test was also carried out on medication groups (Table 2). Pairwise comparison tests using the Dunn test and the Bonferroni correction with the *α* = 0.05 significance level were carried out between pairs of medication groups to ascertain whether taking medicine for mental health influenced levels of retrospective disintegration.

Second, the ‘dynamisms’ score themselves were the object of a multivariate study using the Euclidean distance between data points to understand how each ‘dynamism’ related to each other. A multi-factor Permutational Multivariate Analysis of Variance (PERMANOVA) was conducted using PRIMER-e (v7) with the PERMANOVA+ add-on. Because the items with ‘shame’ and ‘guilt’ did not have a time specification (Supplementary file S2), they were removed from this analysis. The model was based on a Euclidean distance resemblance matrix with Type III (partial) sums of squares. To maintain sufficient degrees of freedom for the residual error (d. f. = 8), the model included all main effects and two-way interactions, while the three-way interaction term was excluded to avoid over-parametrisation. Significance was determined using 999 permutations of residuals under a reduced model.

To investigate ‘Shame’ and ‘Guilt’ a PERMANOVA was conducted with the factors ‘Dynamisms’ and ‘Time’; however, the ‘Dynamisms’ × ‘Time’ interaction could not be estimated due to insufficient residual degrees of freedom.

Finally, to visualise level III ‘dynamisms’, a nMDS was performed. The nMDS was conducted in the software ‘Primer’ with k = 2 dimensions and a maximum of 100 iterations. The analysis was run automatically multiple times with random starting configurations to ensure a global minimum stress solution. The final stress value was assessed to determine the goodness-of-fit of the ordination with a value of 0.2 or less was deemed acceptable.

## Results

Out of 514 survey participants, only 311 finished the survey and only 262 answers were deemed usable. 66% were from female, and the overall age distribution was bimodal with peaks at 28–41 and 61–76 years old. When asked to recall an event that could serve as anchor when answering the survey questions, 87 participants chose an event which related to suicide lived experience, leaving 178 participants with another event.

Events cited by participants without suicide lived experience (control group) related to the loss of a loved one (*n* = 47), relationship breakdown (*n* = 37), distressing medical diagnosis (*n* = 33), the loss of a pet (*n* = 15) and accident to self (*n* = 15). Less frequent event types included financial difficulties (*n* = 6), losing a loved one to suicide (*n* = 7), loss of mental health (*n* = 8) and abusive relationships (*n* = 10).

More than half (58.6%) of the participants had never taken medication for mental health while 9.5% were taking medication for mental health at the time of the survey, 19.5% had taken medication in the past, and 12.4% took medication both in the past and at the time of the survey.

**Figure 1:**
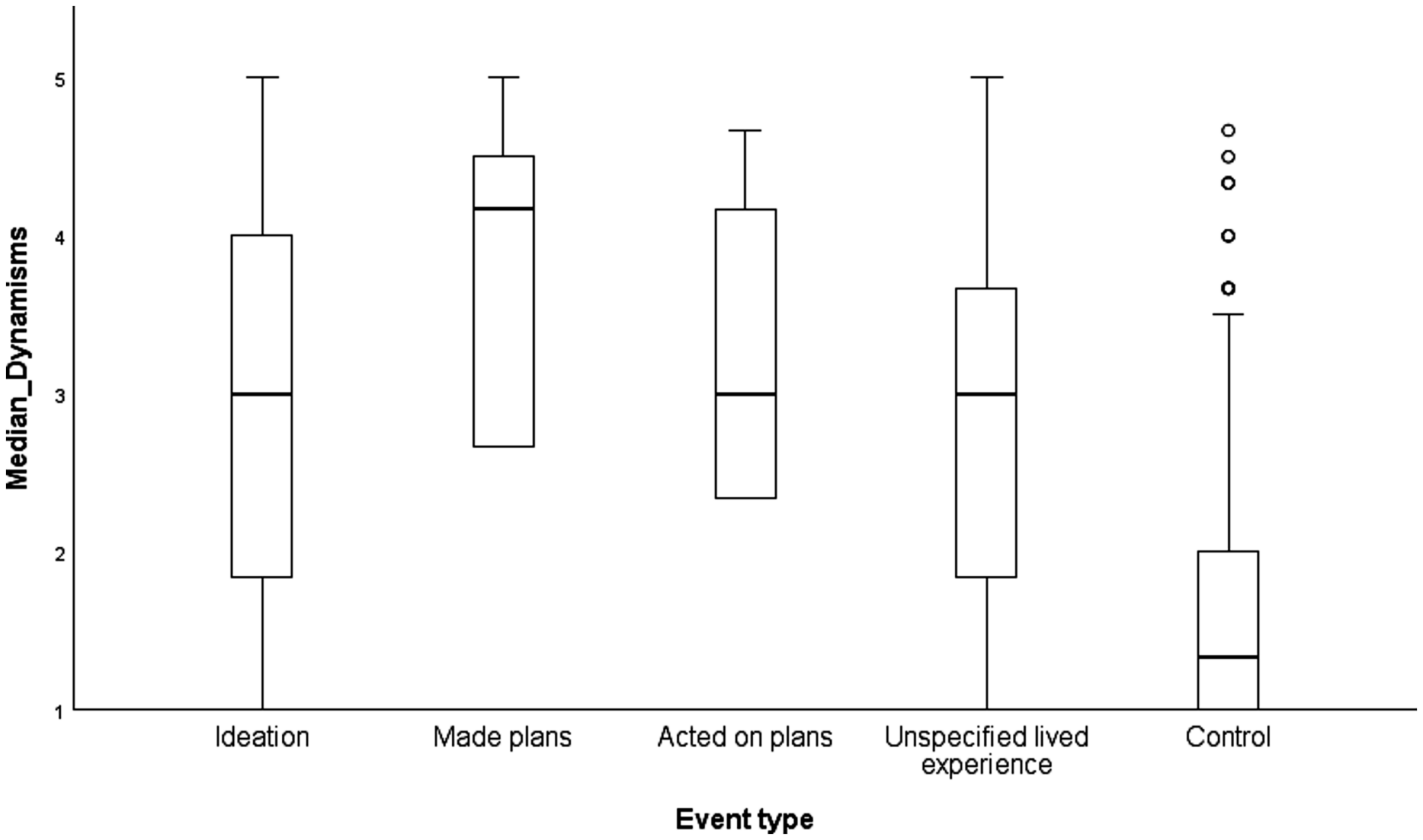
Level III ‘dynamisms’ level across event and lived experience types and control groups.

No significant differences in ‘dynamisms’ scores were found between females and males (*U* = 7063.0; *z* = −0.964; *p* = 0.335) (Supplementary Table S3). A significant difference was found in ‘dynamism’ scores across the event types [H (4) = 52.95, *p* < 0.001] (Supplementary Table S4a). Post-hoc pairwise comparisons revealed that all suicide lived-experience event types (Table 1) had significantly higher mean scores of level III ‘dynamisms’ compared to participants in the control group (*p* < 0.001) (Supplementary Table S4b). Of all participants with suicide lived experience [‘ideated’, ‘planned’, ‘acted on plans’, or ‘unspecified’ (Table 1)], the ‘planned’ group had the highest (4.2) mean disintegrative ‘dynamism’ score (Figure 1).

**Figure 2:**
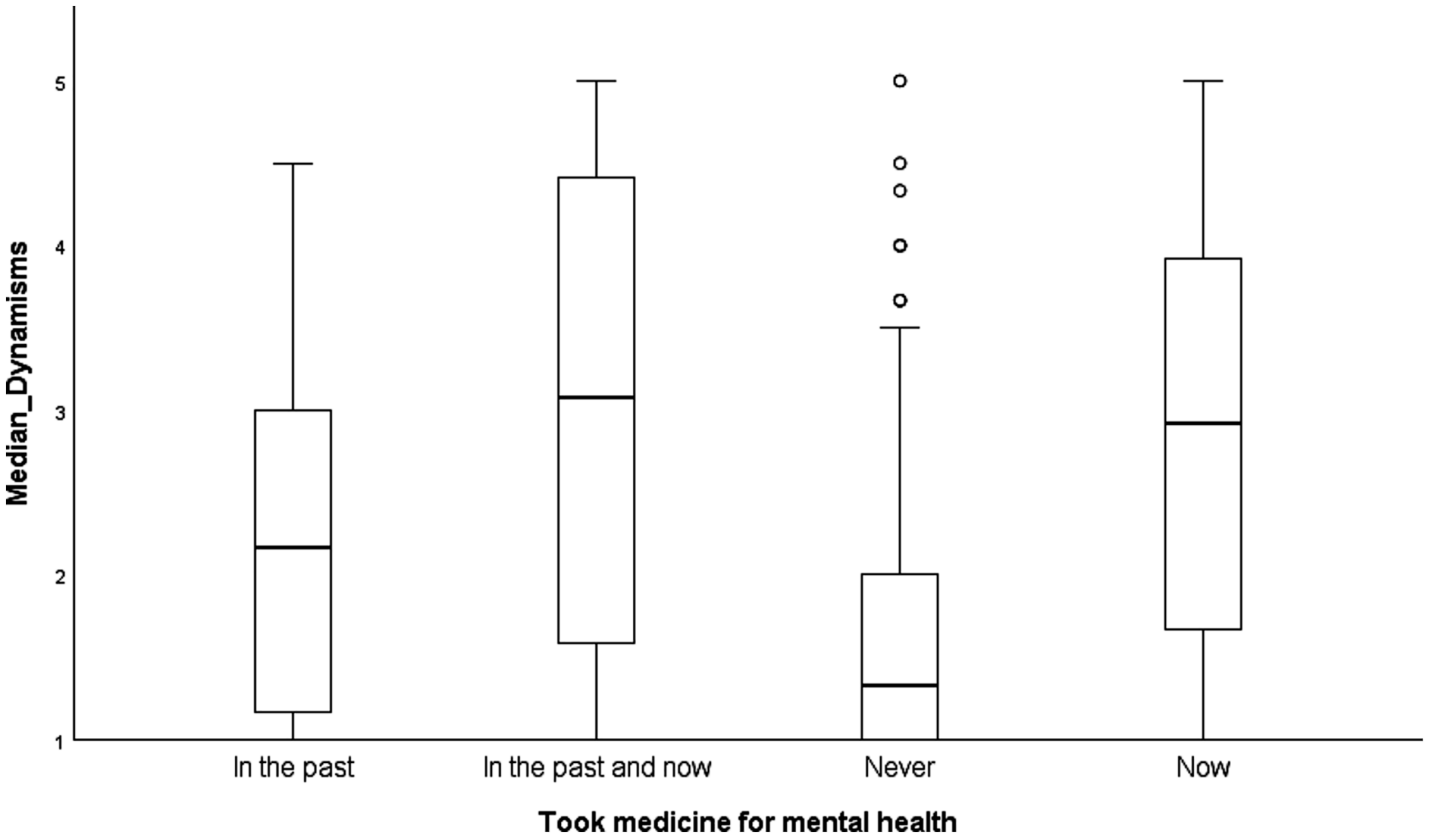
Level III ‘dynamism’ levels across consumption of medication for mental health. Kruskal-Wallis test (*α* = 0.05).

Furthermore, level III disintegrative ‘dynamisms’ were statistically different across the ‘medicine for mental health’ levels [H (3) = 45.44, *p* < 0.001] (Supplementary Table S5a; Figure 2). They were lowest in participants who had never been on medication for mental health (Figure 2) and highest in participants who had not only been on medication in the past but were still on medication at the time of the survey (Figure 2). There were significant differences between the participants who had never been on medication for mental health and participants who had been on medication at any point (Table 2; Supplementary Table S5b).

## PERMANOVA on ‘dynamisms’ (main effects and pairwise tests)

### Main effect and pairwise test

The three-way PERMANOVA (excluding ‘shame’ and ‘guilt’) revealed highly significant main effects for ‘dynamism’ (Pseudo-*F* = 15.53, *p* = 0.001), ‘Location’ (Pseudo-*F* = 4.52, *p* = 0.001), and ‘Time’ (Pseudo-*F* = 18.07, *p* = 0.001) (Supplementary Table S6a). Two-way interactions were also highly significant for “Dynamism’ x Location’ (Pseudo-*F* = 3.70, P(perm) = 0.001) and “‘Dynamism x Time’ (Pseudo-*F* = 2.40, P(perm) = 0.001). The interaction between ‘Location’ and ‘Time’ was not significant [P(perm) = 0.19] (Supplementary Table S6a).

The pairwise test for ‘dynamisms’ (without ‘shame’ and ‘guilt’) revealed that all were different from each other with P(perm) from 0.026 to 0.049 (Supplementary Table S6b). The Average Distance between/within group revealed that ‘Surprised’ (13.09) vs. ‘Inferior’ (12.88) yielded the biggest contrast with the largest t-value (*t* = 5.01) (Supplementary Table S6b). Another strong distinction appeared between ‘Out_proportion’ (16.32) vs. ‘Inferior’ (12.88) (*t* = 4.58) (Supplementary Table S6b). The lowest t-value came from the comparison between ‘Inferior’ vs. ‘Dissatisfied’ (*t* = 2.98), followed by ‘Out_proportion vs. Surprised (*t* = 3.43) (Supplementary Table S6b). The average distance table’s diagonal indicated that ‘inferior’ (12.88) and ‘surprised’ (13.09) had the lowest internal variation (Supplementary Table S6b).

The pairwise test for ‘Location’ revealed that all ‘Locations’ were significantly different to each other [P(perm) 0.016 to 0.022] (Supplementary Table S6c). The t statistic of the pairwise test showed that ‘Behaviour’ and ‘Thoughts’ had the largest difference (*t* = 2.1836), while ‘Thoughts’ and ‘Emotions’ which were more like each other (*t* = 2.0792) (Supplementary Table S6c). ‘Thoughts’ had the lowest within-group average distance value (16.702) (Supplementary Table S6c) making it the ‘Location’ that was scored the most consistently by participants (Supplementary Table S6c).

The pairwise test for ‘Time’ was significant [P(perm) = 0.001] (Supplementary Table S6d). The within group distance matrix indicated that participants scored items with the ‘Year’ timestamp (15.448) more consistently than those with the ‘Months’ timestamp (17.567) (Supplementary Table S6d).

### ‘Shame’ and ‘guilt’ comparison

The ‘Dynamisms’ ‘Shame’ and ‘Guilt’ were tested with a PERMANOVA on their own. The effect of ‘dynamism’ was not significant, Pseudo-*F* (1,1) = 3.37, P(perm) = 0.243 (Supplementary Table S6e). Similarly, the effect of ‘Time’ was non-significant, Pseudo-F (1,1) = 3.09, *p* = 0 0.258 (Supplementary Table S6e).

### nMDS visualisation

A nMDS carried out on the scores of the 8 different ‘dynamisms’ (Table 2) depicted the results of the PERMANOVA, namely that ‘dynamisms’, although similar, are not synonyms (Supplementary Figure S1a), that the location (‘Behaviour’, ‘Thoughts’, ‘Emotions’, ‘N/A’) revealed a low level of within group similarity (Supplementary Figure S1b), and ‘Months’ and ‘Year’ were distinct (Supplementary Figure S1c).

## Discussion

This study, is, to our knowledge, one of the first attempts at measuring retrospective TPD’s disintegrative level III ‘dynamisms’ in a group of participants with suicide lived experience. The low-level disintegration in the control group fits Dąbrowski’s prediction that few individuals have the combination of the three factors [overexcitabilities (genes), environment, and the third factor (~inner drive to better oneself)] necessary for a transition towards level III and above (Dąbrowski, 1964).

The participants who never used medicine to support their mental health had the lowest level III ‘dynamisms’. However, people with a mental health diagnosis are likely to be taking pharmacological support, which may obscure whether using medicine to support a person’s mental health can be equated to reducing levels of disintegration. If disintegration cannot be lowered by medicine, high scores of level III ‘dynamisms’ in a person may be best addressed through counselling with a practitioner with a knowledge of TPD as we know that support through counselling is likely to have an important role to play for the patient’s recovery (Cuijpers et al., 2020).

The study of the ‘dynamisms’ themselves, and how similar they are in what they measure, revealed that they were statistically different from each other and, therefore, cannot be viewed as synonyms (Supplementary Tables S1a, S6a). ‘Dynamisms’ were the primary driver of variation in this model, but their influence was strongly dependent on both the specific location, and the time point of measurement.

The three psychological locations of where ‘dynamisms’ could be at play did matter, and participants scored the Likert items differently depending on whether they were thinking about their ‘Behaviour’, their ‘Emotions’, or their ‘Thoughts’. Of all psychological locations, ‘Thoughts’, showed the least individual variation, indicating that future studies could benefit from focusing on where the ‘Dynamisms’ occurred in participants’ thoughts.

The ‘Dynamisms’ ‘Inferior’ and ‘Surprised’ showed the lowest within-group Euclidean distances. This suggests that participants had a consistent response to items formulated with those ‘Dynamisms’, which indicates that they may be good candidates for future studies.

Unlike other ‘Dynamisms’, ‘Shame’ and ‘Guilt’ were shown to remain present over months and years and may be the best point of entry to use during counselling and when approaching a patient on the topic of past lived experience.

In this study, the sole focus was on the individual psyche, and the ‘Dynamism’ scoring was retrospective, hampering us to conclude that using ‘Dynamisms’ for gauging suicide risk is possible. Also, we know that suicide prediction is complex because of the multitude of proximal and distal risk factors present (Misiak et al., 2023), including a history of mental health struggles (including suicide attempts) (Favril et al., 2022; Sobanski et al., 2021). Furthermore, the cultural reference that comes with TPD may not be applicable universally; so, using principles of TPD in counselling may be helpful in only some circumstances.

While TPD ‘Dynamisms’ offer a developmental lense through which suicidality may carry existential meaning [which is consistent with Pereira’s (2024) argument against purely pathological framings] the recontextualization offered by TPD must not substitute for clinical vigilance, as the intensity of a disintegrative crisis may be a risk factor (Dąbrowski, 1964). According to TPD, when a person does not manage to transition away from the level III into level IV and, instead, stays immobilised at level III, they are said to experience ‘negative disintegration’ (Dąbrowski, 1972a, 1972b). The intense psychological strain of that level can include deeply distressing emotions and profound inner conflict (Dąbrowski, 1964) which might lead some individuals to consider suicide to alleviate their emotional turmoil.

TPD speaks more specifically of two types of mental conditions that are potentially related to disintegration: depression and anxiety (Dąbrowski et al., 1970). At the time of TPD formulation, clinical knowledge of all depression and anxiety types was not as comprehensive as today, so the TPD approach in treating depression and anxiety still needs to be studied and our understanding of its utility refined. The concept of Positive Disintegration appears to align better with adjustment depressive states than to more chronic, clinical depression. Existential depression (Berra, 2019, 2021) when affected patients show a persistent, deep sadness and hopelessness relating to the meaning of life (Berra, 2019) could lend itself well to treatment with TPD guidance. The results of this study are therefore only to be interpreted as exploratory only.

Still, since death by suicide is still a substantial cause of death, particularly in young people (WHO, 2021), every effort at improving our detection is worth attempting as we know that current instruments for the assessment of suicide risk can be improved (Runeson et al., 2017). Level III ‘Dynamisms’ as expressed in TPD offer another avenue for counsellors when working with their patients with suicide lived experience. The findings of this study provide empirical support for TPD ‘Dynamisms’ as a potentially valuable vocabulary for clinicians to explore patients’ inner states, particularly in the context of retrospective accounts of suicidality.

Several limitations should be considered when interpreting the findings of this exploratory study. First, the cross-sectional and retrospective design precludes any causal or predictive inferences regarding the relationship between ‘dynamisms’ and suicidality. Second, reliance on retrospective self-report introduces the possibility of recall bias, particularly in relation to emotionally salient experiences. Third, the use of a distress-based control group rather than a clinically assessed or diagnostically matched comparison group may limit the specificity of group differences. Fourth, the absence of formal diagnostic data restricts the ability to contextualise findings within established clinical categories. Some of the level III ‘Dynamisms’ feature in depression scales like the Beck Depression Inventory (Beck et al., 1996) or specific suicide rating scales like the Columbia-Suicide Severity Rating Scale (CSSRS) (Posner et al., 2011). Finally, the simplified categorisation of the ‘time’ variable may have reduced sensitivity to more nuanced temporal patterns in lived experience.

Further research in this field may try to determine whether there is a ‘window of opportunity’ for patients suffering from disintegration during which they are most likely to be able to turn their anxiety and depression into a transition to level IV and its own ‘dynamisms’, reducing suicide risk.

To conclude, this study is an encouraging step towards an empiric validation of TPD ‘dynamisms’, and further studies could look at the time dimension and the location of the ‘dynamisms’ in more detail while controlling for anxiety/depression and pharmacological support. If further studies manage to show that disintegrative ‘dynamisms’ can help predict suicide risk, they may have their place in the arsenal that health professionals use to gauge suicide risk.

## Data Availability

The raw data supporting the conclusions of this article will be made available by the authors, without undue reservation.
