# Supplementary material for: Using the theory of positive disintegration’s ‘dynamisms’ to gauge retrospective suicide lived experience
Source: Front Psychol. 2026 Jun 17;17:1810469. doi: 10.3389/fpsyg.2026.1810469 (PMC13321191; doi:10.3389/fpsyg.2026.1810469)
Supplement: Supplementary file 1 [file Supplementary_file_1.DOCX]

Supplementary materials

# **Using the “Theory of Positive Disintegration’s dynamisms to gauge retrospective suicide lived experience.**

Marie-Lise Schläppy, Michael Kyron, Susanne Stanley, Dawn Freshwater, Sean Hood

**Tab. S1** Items total, Cronbach’ α, Mean and Standard Deviation (SD) per dynamism, time & location.

| **Scale** | **N items** | **Cronbach's α** | **Mean** | **SD** |
| --- | --- | --- | --- | --- |
| Total scale | 36 | .984 | 72.51 | 36.70 |
| **Dynamisms** |  |  |  |  |
| D_out_proportion | 6 | .932 | 12.44 | 6.73 |
| Guilty | 2 | .907 | 4.13 | 2.53 |
| Shame | 2 | .926 | 3.90 | 2.53 |
| Surprised | 6 | .953 | 11.53 | 6.43 |
| Worried | 6 | .959 | 12.16 | 6.94 |
| Inferior | 6 | .962 | 11.81 | 7.08 |
| Dissatisfied | 6 | .947 | 11.92 | 6.64 |
| Dissatisfied_can_do_better | 2 | .877 | 4.61 | 2.71 |
| **Months items** | 18 | .969 | 37.42 | 19.06 |
| **Years items** | 18 | .976 | 35.09 | 18.83 |
| **Behaviour items** | 10 | .937 | 19.74 | 10.07 |
| **Thoughts items** | 10 | .948 | 19.77 | 10.20 |
| **Emotions items** | 10 | .946 | 20.36 | 10.51 |

**Tab. S2** Exploratory Factor Analysis Total Scale (D= Dynamism, behav=behaviour, thou=thoughts, emo=emotions, mo=months, yr=years). Principal Axis Factoring method. 4 factors extracted, 6 iterations required.

| **Counter** | **Item** | **Factor 1 Loading** | **Factor 2 Loading** | **Factor 3 Loading** | **Factor 4 Loading** |
| --- | --- | --- | --- | --- | --- |
| 1 | D_out_proportion_behav_mo | 0.618 | 0.324 | 0.093 | 0.310 |
| 2 | D_out_propotion_behav_yr | 0.686 | 0.330 | -0.226 | 0.254 |
| 3 | D_out_propotion_emo_mo | 0.682 | 0.281 | 0.232 | 0.417 |
| 4 | D_out_propotion_emo_yr | 0.783 | 0.270 | -0.246 | 0.283 |
| 5 | D_out_propotion_thou_mo | 0.724 | 0.377 | 0.174 | 0.328 |
| 6 | D_out_propo_thou_yr | 0.778 | 0.379 | -0.201 | 0.215 |
| 7 | Guilty_mo | 0.789 | -0.058 | 0.181 | -0.043 |
| 8 | Guilty_yr | 0.811 | -0.051 | -0.123 | 0.012 |
| 9 | Shame_mo | 0.828 | -0.107 | 0.123 | -0.044 |
| 10 | Shame_yr | 0.840 | 0.001 | -0.135 | -0.003 |
| 11 | Surprised_behav_mo | 0.802 | 0.274 | 0.206 | -0.174 |
| 12 | Surprised_behav_yr | 0.812 | 0.271 | -0.167 | -0.101 |
| 13 | Surprised_emo_mo | 0.720 | 0.341 | 0.276 | -0.233 |
| 14 | Surprised_emo_yr | 0.781 | 0.364 | -0.188 | -0.256 |
| 15 | Surprised_thou_mo | 0.770 | 0.313 | 0.279 | -0.204 |
| 16 | Surprised_thou_yr | 0.809 | 0.290 | -0.144 | -0.240 |
| 17 | Worried_behav_mo | 0.806 | 0.069 | 0.283 | -0.195 |
| 18 | Worried_behav_yr | 0.848 | 0.142 | -0.182 | -0.262 |
| 19 | Worried_emo_mo | 0.871 | -0.050 | 0.232 | -0.088 |
| 20 | Worried_emo_yr | 0.867 | 0.003 | -0.185 | -0.159 |
| 21 | Worried_thou_mo | 0.836 | -0.009 | 0.163 | -0.109 |
| 22 | Worried_thou_yr | 0.843 | 0.045 | -0.184 | -0.135 |
| 23 | Inferior_behav_mo | 0.821 | -0.282 | 0.007 | 0.069 |
| 24 | Inferior_behav_yr | 0.826 | -0.249 | -0.251 | 0.065 |
| 25 | Inferior_emo_mo | 0.805 | -0.310 | 0.059 | -0.015 |
| 26 | Inferior_emo_yr | 0.816 | -0.244 | -0.245 | -0.040 |
| 27 | Inferior_thou_mo | 0.792 | -0.333 | 0.060 | 0.066 |
| 28 | Inferior_thou_yr | 0.827 | -0.292 | -0.189 | 0.052 |
| 29 | Dissatisfied_behav_mo | 0.756 | -0.276 | 0.285 | 0.001 |
| 30 | Dissatisfied_behav_yr | 0.797 | -0.255 | -0.151 | -0.014 |
| 31 | Dissatisfied_emo_mo | 0.842 | -0.206 | 0.269 | 0.089 |
| 32 | Dissatisfied_emo_yr | 0.852 | -0.160 | -0.195 | 0.079 |
| 33 | Dissatisfied_thou_mo | 0.837 | -0.223 | 0.241 | 0.053 |
| 34 | Dissatisfied_thou_yr | 0.860 | -0.200 | -0.157 | -0.004 |
| 35 | Dissatisfied_candobetter_mo | 0.741 | -0.230 | 0.261 | 0.182 |
| 36 | Dissatisfied_candobetter_yr | 0.804 | -0.237 | -0.123 | 0.074 |
|  |  | **Factor 1 loading range** |  |  |  |
| X | **Years items** | 0.686 - 0.867 |  |  |  |
| X | **Months items** | 0.618 - 0.871 |  |  |  |
| X | **Behaviour items** | 0.686 – 0.848 |  |  |  |
| X | **Thoughts items** | 0.724 - 0.860 |  |  |  |
| X | **Emotions items** | 0.682 - 0.867 |  |  |  |

**Tab. S3** Mann-Whitney test on Dynamisms scores on gender (Female or Male)

|  | **Median_Dynamisms** |
| --- | --- |
| U of Mann-Whitney | 7063.000 |
| W of Wilcoxon | 10891.000 |
| Z | -0.964 |
| Sig. asymptotic (bilateral) | 0.335 |

**Tab. S4a** Kruskal-Wallis H test on event type (k=5, n=262)

| Test | Statistic (H) | df | p-value | Effect size |
| --- | --- | --- | --- | --- |
| Kruskal-Wallis | H=52.95 | 4 | <.001 | η2=.18 |

**Tab. 4b** *Pairwise comparisons of disintegration level between event types.*

** Indicates significance* *(α = 0.05)*

| **Event type 1-**  **Event type 2** | **Standardised Test Statistic (Z)** | **Significance level** |  |
| --- | --- | --- | --- |
| Control-Unspecified * | 71.09 | <0.001 |  |
| Control- Ideation * | 76.89 | <0.001 |  |
| Control-Acted* | 98.74 | 0.009 |  |
| Control-Planned* | 117.41 | <0.001 |  |
| Unspecified - Ideation | 5.80 | 0.727 |  |
| Unspecified - Acted | 27.65 | 0.480 |  |
| Unspecified - Planned | 46.32 | 0.191 |  |
| Ideation -Acted on Plans | -21.85 | 0.578 |  |
| Ideation- Made Plans | -40.53 | 0.253 |  |
| Acted on Plans - Planned | 18.67 | 0.710 |  |
| Each row tests the null hypothesis that the Event type 1 and Event type 2 distributions are the same.  Asymptotic significances (2-sided tests) are displayed. The significance level is α = 0.05. | | | |
| a) Significance values have been adjusted by the Bonferroni correction for multiple tests. | | | |

**Tab. S5 a** Kruskal-Wallis H test on medicine for mental health (k=4, n=262)

| Test | Statistic (H) | df | p-value | Effect size |
| --- | --- | --- | --- | --- |
| Kruskal-Wallis | H=45.44 | 3 | <.001 | η2= .17 |

**Tab. S5 b** *Pairwise comparisons of groups relating to the consumption of medicine for mental health. Pairwise comparison between participants on mental health medication now, in the past or both and participants without medication support in relation to average level III dynamism score. * Indicates significance (α = 0.05)*

| **Never-Medicine** | **Standardised Test Statistic (Z)** | **Std. Error** | **Std. Test**  **Statistic** | **Sig.** | **Adj. Sig.^a^** |
| --- | --- | --- | --- | --- | --- |
| Never - In the past* | 35.576 | 12.236 | 2.907 | .004 | .022 |
| Never - Now* | -72.206 | 16.461 | -4.386 | <.001 | .000 |
| Never - In the past & now* | 75.669 | 13.331 | 5.676 | <.001 | .000 |
| In the past- Now | -36.630 | 18.577 | -1.972 | .049 | .292 |
| In the past- In the past & now | -40.093 | 15.869 | -2.526 | .012 | .069 |
| Now- In the past & now | 3.463 | 19.315 | .179 | .858 | 1.00 |

Each row tests the null hypothesis that the Sample 1 and Sample 2 distributions are the same.

Asymptotic significances (2-sided tests) are displayed. The significance level is α = 0.050.

a. Significance values have been adjusted by the Bonferroni correction for multiple tests.

**Tab. S6** PERMANOVA & pairwise tests

1. Main test


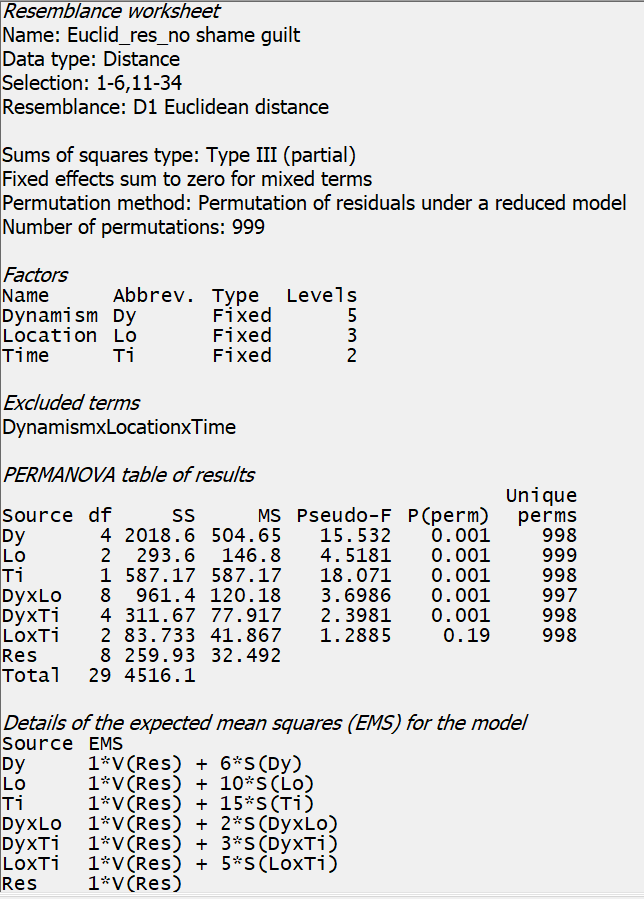


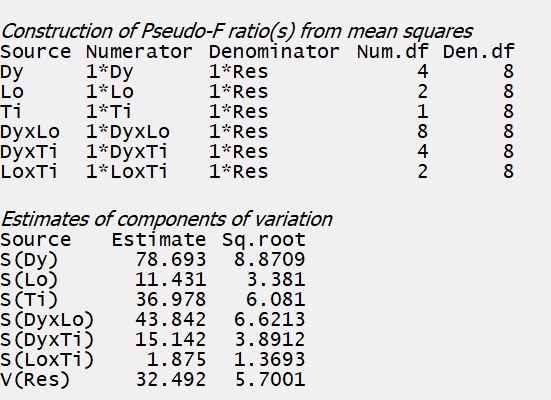


1. Pairwise test for ‘dynamisms’ without ‘shame’ and ‘guilt’


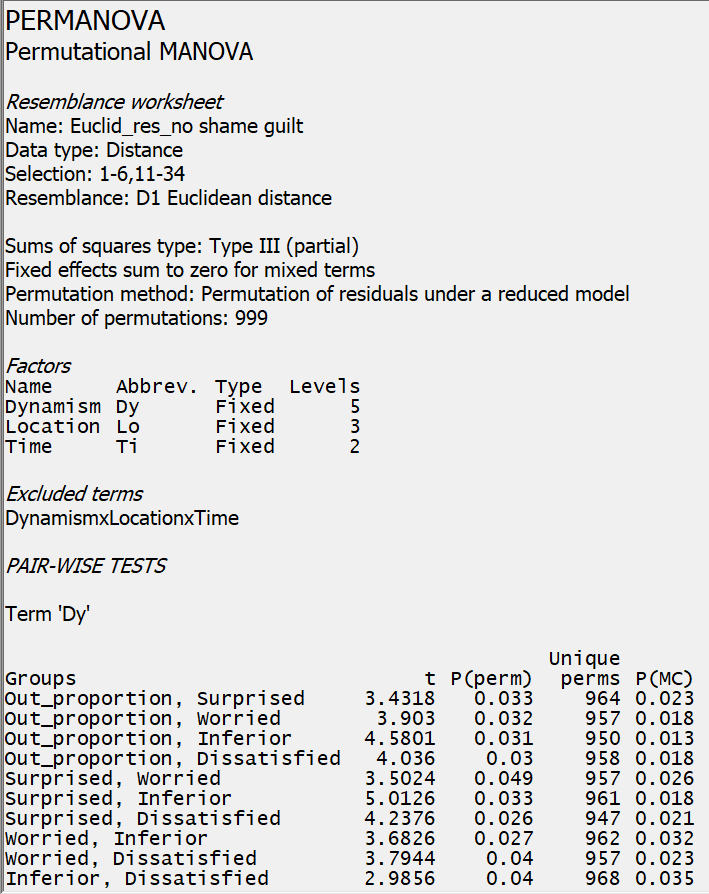


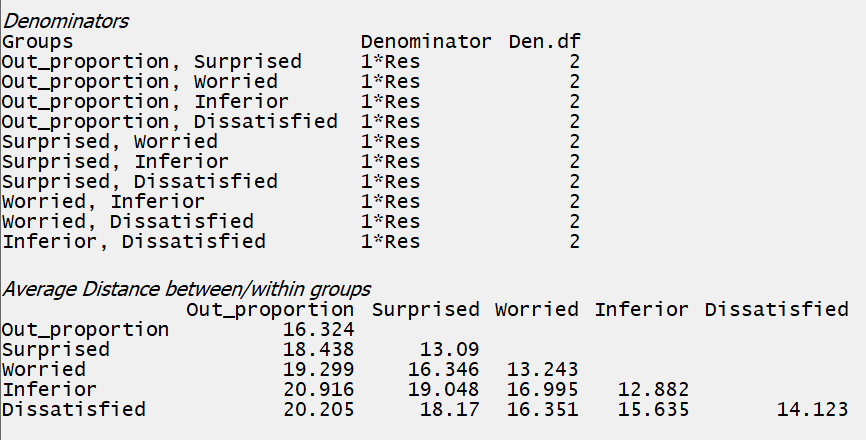


1. Pairwise test for location’ without ‘shame’ and ‘guilt’


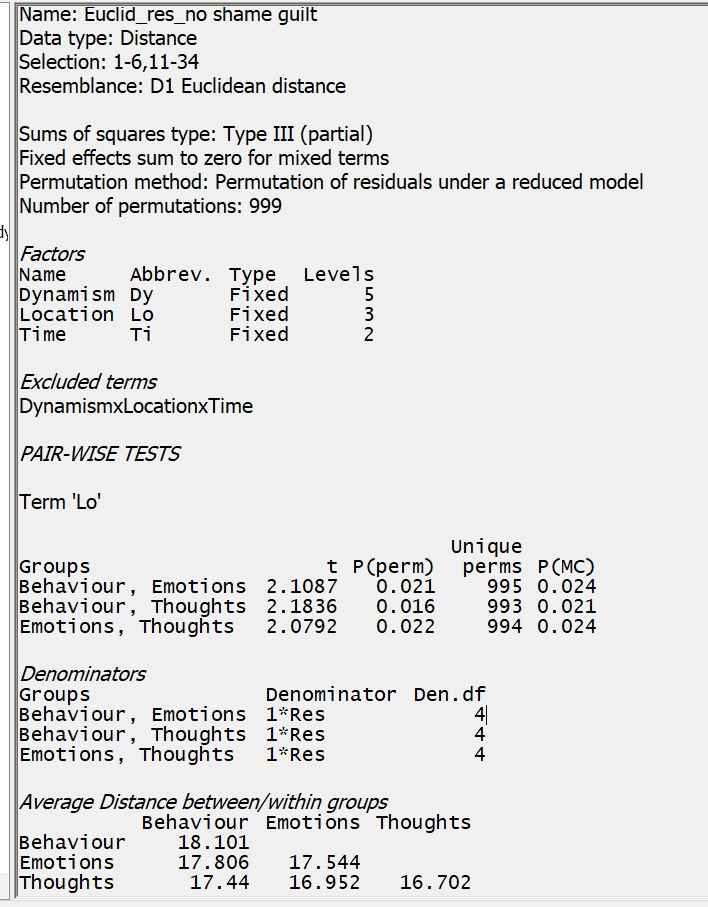


1. Pairwise for ‘time’ without ‘shame’ and ‘guilt’


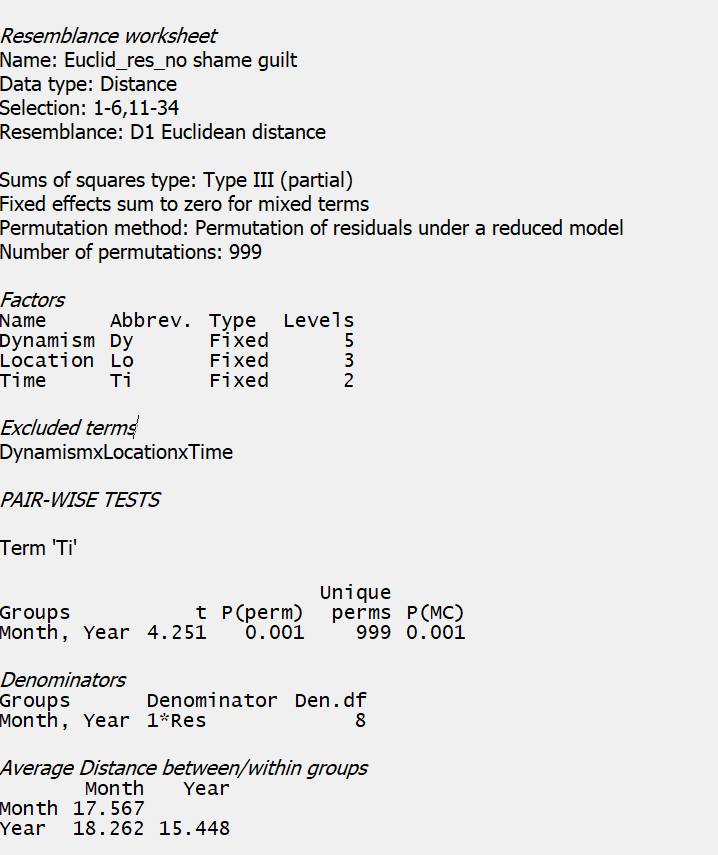


1. Main effect for ‘shame’ and ‘guilt’ (whose items did not have a location, but only time)
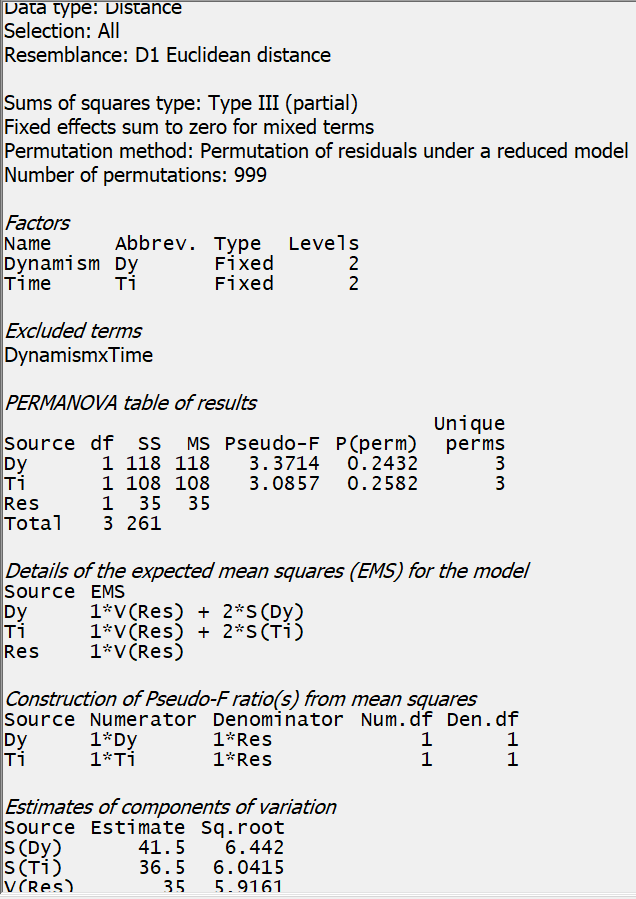


**Fig. S1 Non-metric Multidimensional Scaling (nMDS)**

1. Dynamisms


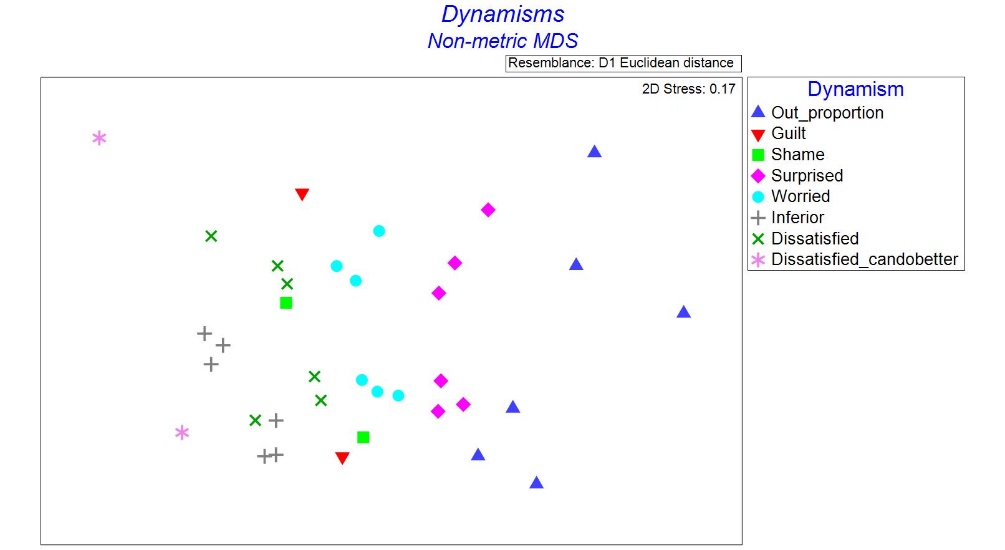


***Fig. S1a*** *Non-metric multi-dimensional scaling (nMDS) based on the Euclidean distance between dynamism types.*

1. Location


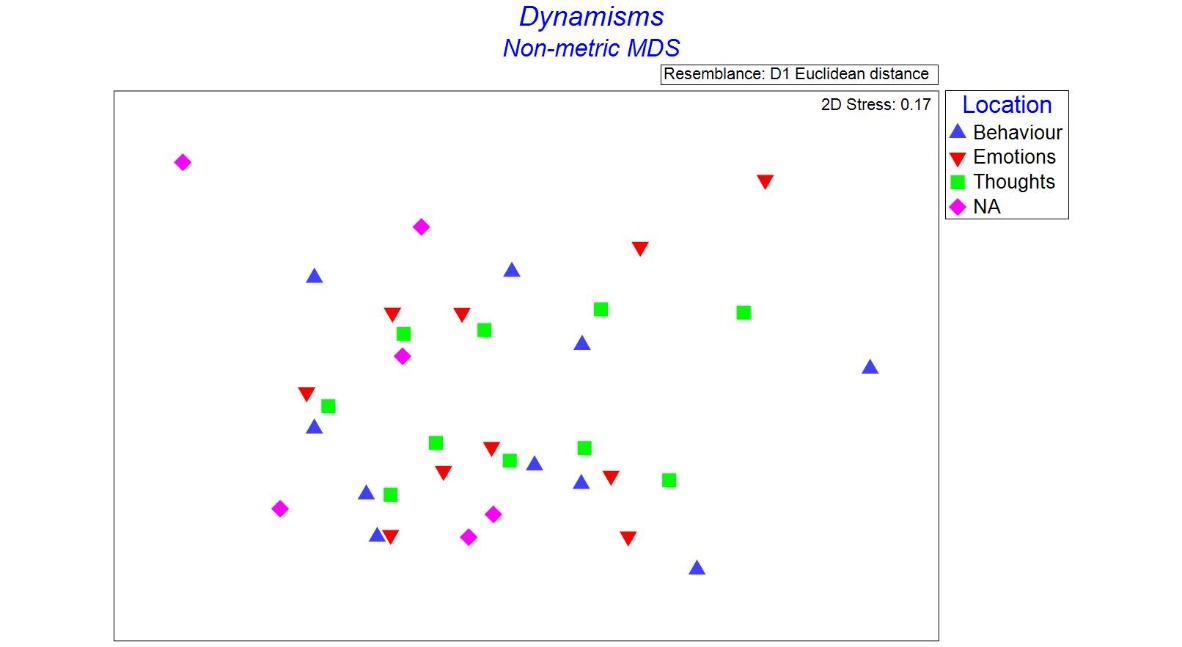


***Fig. S1b*** *Non-metric multi-dimensional scaling (nMDS) based on the Euclidean distance between the location of where the dynamisms were at play (i.e. behaviour, emotion, and thoughts, or overall (NA)).*

1. Time


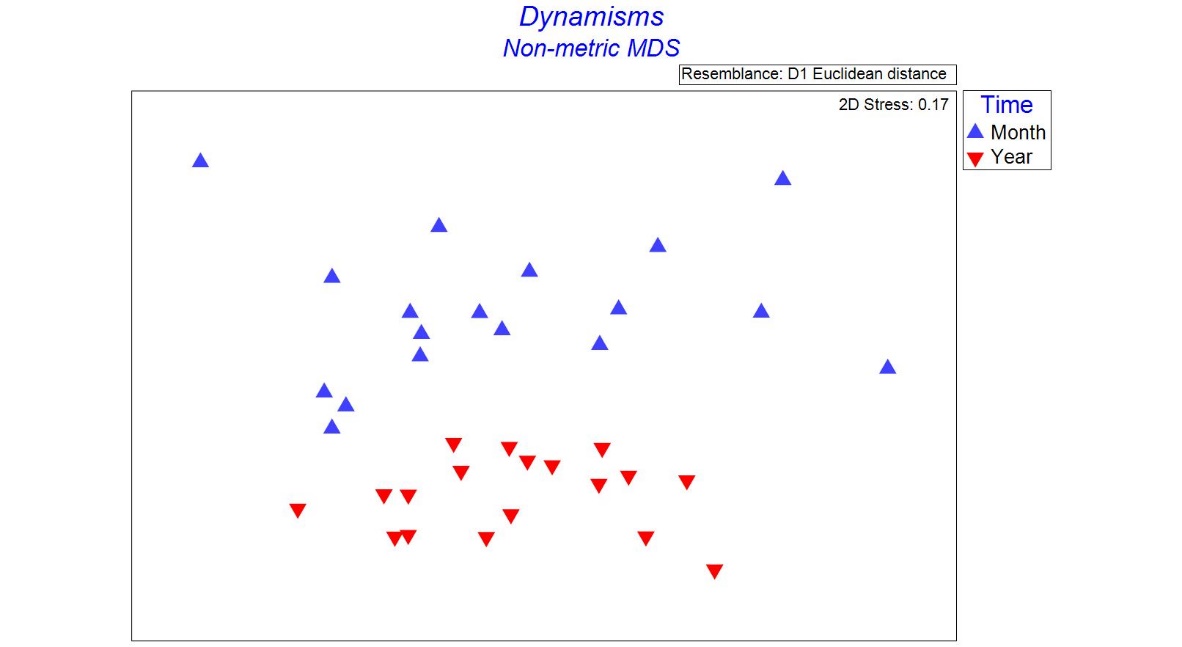


***Fig. S1c*** *Non-metric multi-dimensional scaling (MDS) based on the Euclidean distance between the time at which the dynamisms were at play (i.e. months or years before the event/lived experience).*
