# Supplementary material for: Using the theory of positive disintegration’s ‘dynamisms’ to gauge retrospective suicide lived experience
Source: Front Psychol. 2026 Jun 17;17:1810469. doi: 10.3389/fpsyg.2026.1810469 (PMC13321191; doi:10.3389/fpsyg.2026.1810469)
Supplement: Supplementary file 2 [file Supplementary_file_2.pdf]

Informed consent

Participant Information Form

Project title: Evaluating the potential for the dynamisms of the theory of positive disintegration to function as a suicide developmental vulnerability index.

UWA School of Medicine / Division of Psychiatry, M521 The University of Western Australia
35 Stirling Highway, Crawley WA 6009
Tel: 08 6457 2393
Email: tpd-project@uwa.edu.au

Invitation:

You are invited to participate in a project that is trying to find ways to prevent suicide. Unfortunately, suicide is still the main cause of mortality amongst young people, and too often occurs in older people also. The research team in this project wants to see if information contained in a psychological theory, called the “Theory of Positive

Disintegration” by K. Dabrowski, can offer some insights to detect when people are undergoing “disintegration” -which could help detect when people need help.

We are distributing the survey to a large number of people to compare how the general public scores in the survey compared to people who have experienced suicidal thoughts, have made suicide plans or who have tried to take their life.

In this survey we ask you to think of an “event” and to answer the questions while keeping this “event” in mind. For those of you, who have had suicidal thoughts, have planned a suicide or who have acted on those plans, please think of that as your event. For those of you who have not experienced a suicide-related event please think of another actual event that made a great impression on you and caused you uncomfortable thoughts or painful emotions (e.g. having a car accident, being diagnosed with a serious disease, losing a loved one, taking a big test, parents divorcing, homelessness, a relationship break up, the loss of a much loved pet or another similar event).

In this online survey we would like to know your thoughts, feelings and experiences in the weeks and years leading up to your event.

Aim of the study (What is the project about?)

The aims of this study are:

1. To find out whether certain characteristics are the same in the general population compared to people who think of, plan for, or try to take their own life;

2. To find out whether certain behaviours, emotions and thoughts emerge weeks and years before a specific event, and whether they are the same for people in the general population compared to people who think of, plan for, or try to take their own life.

3. To identify which concepts of the theory of positive disintegration could become part of a developmental vulnerability index to help people with suicidal thoughts.

What does participation involve?

To help those who will experience these issues in the future, it is important that we have a good understanding of the experiences that people go through. Participation involves completing an de-identified online survey that will take approximately 40 minutes to complete. During the survey you will be asked how you felt prior to a certain actual event. For those of you who have thoughts about suicide, made plans or acted on those plans, the event to refer to during the survey will be your suicide thoughts, plans or attempt. For others, you will be asked to answer the questions while thinking of an event that made a big impression on you and caused negative emotions or distress.

Voluntary participation and withdrawal from the study

Participation in this investigation is entirely voluntary. Participants can withdraw from the survey at any time, without giving an explanation.

Your privacy

All data will be stored in a de-identified format, in a password-protected computer or a secure server for a minimum of seven years, after which the data will be deleted. This project and survey instrument have been approved by the UWA human ethics committee.

How will the results be used?
The de-identified results from this investigation will be disseminated including through academic publications, at conferences and via (social) media. If you want to read the publication that will be published in a specialized journal when it comes out, please email tpd-project@uwa.edu.au.

Possible Benefits

The study will provide valuable information about how to prevent suicide. It may help address suicide risk, which remains a very worrying public health problem.

There are possible personal benefits from participating in this study. For example, the questions in the survey allow you to learn more about yourself. You might also identify any unresolved issues that you may have with your experiences, past and present, and find that it would be a good idea to get help with those issues. An appropriate helpline and support information is provided at the end of the survey if you want to take this further.

Because the questions in this study ask you to reflect on your past and associated feelings, you might find some questions uncomfortable or even distressing. In this case, you can stop the survey and return to it later. If that happens, you will receive an email asking you a week later if you are willing to finish the survey. There will also be helpline phone numbers

available if you need to speak to someone.

Compensation

If you withdraw from the study, you will not receive any compensation.

Funding source
The funding for this stud [illegible]
[illegible] ity grant awarde [illegible]
[illegible] Details about this Foundation can be viewed at
https://www.uwa.edu.au/institutes/young-lives-matter/home

Contacts

[illegible] ny aspect of this study please feel free to contact
[illegible] or tpd-project@uwa.edu.au.

Sincerely,

Approval to conduct this research has been provided by the University of Western Australia, in accordance with its ethics review and approval procedures. Any person considering participation in this research project, or agreeing to participate, may raise any questions or issues with the researchers at any time. In addition, any person not satisfied with the response of researchers may raise ethics issues or concerns, and may make any

complaints about this research project by contacting the Human Ethics office at UWA on (08) 6488 3703 or by emailing to humanethics@uwa.edu.au. All research participants are entitled to retain a copy of any Participant Information Form and/or Participant Consent Form relating to this research project.

I agree to participate in the survey. By selecting "yes, I consent" and proceeding with this survey, I signify my consent to participate.

Yes, I consent

No, I do not consent

Defining the "event"

In this survey, we ask you to think of an 'event' and to answer the survey questions while referring to this event in your mind.

For those of you who have had suicidal thoughts, have planned a suicide or have acted on those plans please think of that, as your event.

For those of you who did not experience that, please think of another event that made a great impression on you and caused you uncomfortable thoughts or painful emotions (e.g. I had a car accident, I was diagnosed with a serious disease, a loved-one died, I took a big test, my parents divorced, my relationship broke-up or any other event).

In this online survey, we would also like to know your thoughts, feelings and behaviours in the weeks and years leading up to your event.

My event relates to suicide (suicidal thoughts, making plans or acting on the plans)

My even relates to something else

Describe the event in succinct words:

(e.g. I thought about ending my life, I had a car accident, I was diagnosed with a serious disease, a loved-one died, I took a big test, my parents divorced, I became homeless, my relationship broke-up, I lost a much loved pet, or another similar event).

How long ago did this occur?

Participant info

When were you born?

I identify my gender as:

Male

Female

Non-binary / third gender

Prefer not to say

Which country do you live in?

Are you now on medication for mental health, or have you been in the past?

In the past

Now

In the past and now

No

What is your mental health diagnosis now?

What was your mental health diagnosis in the past?

What medication do/did you take in relation to the diagnosis?

Welfare check

The rest of this survey is about how you felt and thought in the past, in relation to your nominated "event". This may be distressing for some people. In this light, do you feel that you can go on with taking the survey NOW?

Yes

No

Dynamisms

Recalling how it was before the event, how do the following statements apply to you?

Before the event I felt that my BEHAVIOUR was out of proportion to the situation I was in.

	Not at all	Somewhat	Moderately	Mostly	Completely
How true in the month before the event?	○	○	○	○	○
How true years before the event?	○	○	○	○	○

Before the event, I felt that my EMOTIONS were out of proportion to the situation I was in.

	Not at all	Somewhat	Moderately	Mostly	Completely
How true in the month before the event?	○	○	○	○	○
How true years before the event?	○	○	○	○	○

Before the event, I felt that my THOUGHTS were out of proportion to the situation I was in.

	Not at all	Somewhat	Moderately	Mostly	Completely
How true in the month before the event?	○	○	○	○	○
How true years before the event?	○	○	○	○	○

Before the event I felt GUILTY about my own behaviour, emotions and/or thoughts.

	Not at all	Somewhat	Moderately	Mostly	Completely
How true in the month before the event?	○	○	○	○	○

	Not at all	Somewhat	Moderately	Mostly	Completely
How true years before the event?	○	○	○	○	○

Before the event I felt SHAME about my own behaviour, emotions and/or thoughts.

	Not at all	Somewhat	Moderately	Mostly	Completely
How true in the month before the event?	○	○	○	○	○
How true years before the event?	○	○	○	○	○

Before the event I felt repeatedly surprised with my BEHAVIOUR.

	Not at all	Somewhat	Moderately	Mostly	Completely
How true in the month before the event?	○	○	○	○	○
How true years before the event?	○	○	○	○	○

Before the event I felt repeatedly surprised with my EMOTIONS.

	Not at all	Somewhat	Moderately	Mostly	Completely
How true in the month before the event?	○	○	○	○	○
How true years before the event?	○	○	○	○	○

Before the event I felt repeatedly surprised with my THOUGHTS.

	Not at all	Somewhat	Moderately	Mostly	Completely
How true in the month before the event?	○	○	○	○	○
How true years before the event?	○	○	○	○	○

Before the event I felt repeatedly worried about my BEHAVIOUR.

	Not at all	Somewhat	Moderately	Mostly	Completely
How true in the month before the event?	○	○	○	○	○
How true years before the event?	○	○	○	○	○

Before the event I felt repeatedly **worried** about my EMOTIONS.

	Not at all	Somewhat	Moderately	Mostly	Completely
How true in the month before the event?	○	○	○	○	○
How true years before the event?	○	○	○	○	○

Before the event I felt repeatedly **worried** about my THOUGHTS.

	Not at all	Somewhat	Moderately	Mostly	Completely
How true in the month before the event?	○	○	○	○	○
How true years before the event?	○	○	○	○	○

Before the event I felt that I was **inferior** to others. This was because I felt that my BEHAVIOUR was inferior compared to the behaviour of others.

	Not at all	Somewhat	Moderately	Mostly	Completely
How true in the month before the event?	○	○	○	○	○

	Not at all	Somewhat	Moderately	Mostly	Completely
How true years before the event?	○	○	○	○	○

Before the event I felt that I was inferior to others. This was because I felt that my EMOTIONS were inferior compared to the emotions of others.

	Not at all	Somewhat	Moderately	Mostly	Completely
How true in the month before the event?	○	○	○	○	○
How true years before the event?	○	○	○	○	○

Before the event I felt that I was inferior to others. This was because I felt that my THOUGHTS were inferior compared to the thoughts of others.

	Not at all	Somewhat	Moderately	Mostly	Completely
How true in the month before the event?	○	○	○	○	○
How true years before the event?	○	○	○	○	○

Before the event I felt dissatisfied with my BEHAVIOUR.

	Not at all	Somewhat	Moderately	Mostly	Completely
How true in the month before the event?	○	○	○	○	○
How true years before the event?	○	○	○	○	○

Before the event I felt dissatisfied with my EMOTIONS.

	Not at all	Somewhat	Moderately	Mostly	Completely
How true in the month before the event?	○	○	○	○	○
How true years before the event?	○	○	○	○	○

Before the event I felt dissatisfied with my THOUGHTS.

	Not at all	Somewhat	Moderately	Mostly	Completely
How true in the month before the event?	○	○	○	○	○

	Not at all	Somewhat	Moderately	Mostly	Completely
How true years before the event?	○	○	○	○	○

Before the event I felt dissatisfied with MYSELF. This is because I know that I should be able to act, feel and think in ways that better represent who I am, but was not able to then.

	Not at all	Somewhat	Moderately	Mostly	Completely
How true in the month before the event?	○	○	○	○	○
How true years before the event?	○	○	○	○	○

Space to expand on the last question (optional)

Consider the following statement: My behaviour, emotions and/or thoughts changed after the event. Please describe succinctly:

My BEHAVIOUR changed in the following way:
(e.g. after my dog passed away I stopped going for walks, or after my suicide attempt I started meditating to help myself through the day).

My EMOTIONS changed in the following way:
(e.g. after my dog passed away I became more compassionate with people whose pets passed away, or after my suicide attempt I felt more grateful to be alive).

My THOUGHTS changed in the following way:
(e.g. after my dog passed away, or after my suicide attempt I became more philosophical about life).

These changes were

Gradual

Abrupt

No changes

Space to expand on last question (optional)

Welfare check 2

In this section we want to make sure that you have not been rattled too much by the questions in this survey.

I feel that I can continue with the rest of my day as planned, even though thinking about some questions might have been uncomfortable.

Yes

No or not sure, I will use strategies that I have used in the past to enable me to cope with distress

Other comments on continuing with the rest of my day as planned:

I have some strategies that I can use to enable me to get through distressing times (for example, if I am distressed now).

Talk to a friend

Talk to a family member

Call Mental Health Helpline

Other

Other strategies that I can use are:

I have additional supports that are accessible to me now:

My doctor

My friends and family

Mental Health Helpline

Other (consider expanding on this below)

Space for expanding on additional support:

Other comments

Other comments about this survey that I would like to add:

Thank you for completing the survey. We hope you are in good spirits now and can continue your day as planned.

If not, please contact suicide prevention help lines in your country.

In Australia you can call the following services for assistance:

Lifeline: 13 11 14
Suicide call back: 1 300 659 467

If you would like to take part in future studies on this topic, please email us at **tpd-project@uwa.edu.au** .

Thank you.

Powered by Qualtrics
